# Identification and Expressional Analysis of Putative PRDI-BF1 and RIZ Homology Domain-Containing Transcription Factors in *Mulinia lateralis*

**DOI:** 10.3390/biology12081059

**Published:** 2023-07-27

**Authors:** Feng Zhao, Xiaolin Guo, Xixi Li, Fang Liu, Yifan Fu, Xiaohan Sun, Zujing Yang, Zhifeng Zhang, Zhenkui Qin

**Affiliations:** 1 Ministry of Education Key Laboratory of Marine Genetics and Breeding, College of Marine Life Sciences, Ocean University of China, Qingdao 266003, China; zhaofeng9785@163.com (F.Z.); 15053868632@163.com (X.G.); lclixixi@163.com (X.L.); lfliufanglf@163.com (F.L.); 18271206112@163.com (Y.F.); sunxiaohan1008@163.com (X.S.); yzj@ouc.edu.cn (Z.Y.); zzfp107@ouc.edu.cn (Z.Z.); 2 Key Laboratory of Tropical Aquatic Germplasm of Hainan Province, Sanya Oceanographic Institution, Ocean University of China, Sanya 572000, China

**Keywords:** *Mulinia lateralis*, PRDM gene family, PGCs, expression profiling

## Abstract

**Simple Summary:**

The formation of primordial germ cells (PGCs) is the basis of molluskan reproduction, but limited information is reported on this topic. PRDI-BF1 and RIZ homology domain-containing proteins (PRDMs), especially PRDM1 (also known as BLIMP1) and PRDM14, have been reported to be essential for the formation of PGCs. In this study, we systematically characterized the putative PRDMs in a bivalve mollusk species, *Mulinia lateralis*, and analyzed their sequence structures, phylogenetic relationships, and expressional profiles. Furthermore, we analyzed the temporal–spatial expression patterns of *Ml-prdm1* and *Ml-prdm14* RNA in early embryos and larvae. Our study suggests that *Ml-prdm1* may function as an important regulator of PGC formation in *M. lateralis*.

**Abstract:**

Mollusca represents one of the ancient bilaterian groups with high morphological diversity, while the formation mechanisms of the precursors of all germ cells, primordial germ cells (PGCs), have not yet been clarified in mollusks. PRDI-BF1 and RIZ homology domain-containing proteins (PRDMs) are a group of transcriptional repressors, and PRDM1 (also known as BLIMP1) and PRDM14 have been reported to be essential for the formation of PGCs. In the present study, we performed a genome-wide retrieval in *Mulinia lateralis* and identified 11 putative PRDMs, all of which possessed an N-terminal PR domain. Expressional profiles revealed that all these *prdm* genes showed specifically high expression levels in the given stages, implying that all PRDMs played important roles during early development stages. Specifically, *Ml-prdm1* was highly expressed at the gastrula stage, the key period when PGCs arise, and was specifically localized in the cytoplasm of two or three cells of blastula, gastrula, or trochophore larvae, matching the typical characteristics of PGCs. These results suggested that *Ml-prdm1*-positive cells may be PGCs and that *Ml-prdm1* could be a candidate marker for tracing the formation of PGCs in *M. lateralis*. In addition, the expression profiles of *Ml-prdm14* hinted that it may not be associated with PGCs of *M. lateralis*. The present study provides insights into the evolution of the PRDM family in mollusks and offers a better understanding of the formation of PGCs in mollusks.

## 1. Introduction

Germline cells transmit genetic and epigenetic information across generations in all sexually reproducing animals to ensure the survival of the species [1,2]. Primordial germ cells (PGCs) that separated from somatic cells during early embryogenesis are common precursors of both sperms and eggs [3,4], and the segregating of germ cells from somatic cells is the first diversification step in the evolution of metazoans [5]. Under the initial determination of germ cell fate, there are two generally accepted theories of PGC specification: preformation, in which the specification of PGCs is determined by maternally inherited determinants in early development, and epigenesis, in which PGCs arise by inductive signals in late development [3,6]. These two mechanisms are not mutually exclusive and are inevitably used simultaneously at a certain stage of germ cell development [6]. So far, the PGC specification modes are different among different species, and both theories are reported in mollusks [7,8,9,10].

PRDM proteins comprise an N-terminal PR (PRDI-BF1 and RIZ1 homology) domain derived from the SET (suppressor of variegation 3–9, enhancer of zeste, and trithorax) domain and a variable number of C-terminal Zn-finger repeats [11,12]. As important transcription factors, PRDMs modulate different cellular processes, including the regulation of germ cell development. For example, PRDM1 and PRDM14 are essential for PGC development in several species. Notably, PRDM1 is functionally conserved across vertebrates and invertebrates. In mice (*Mus musculus*), PRDM1 and PRDM14 have been identified as the key regulators of PGC specification. PRDM1 is a key transcriptional regulator and is primarily responsible for the repression of somatic genes in PGCs [13,14]. The deletion of *prdm1* led to the failure of normal proliferation and migration of mouse PGCs [13], and *prdm1* homozygous mutants lacked PGCs [15]. PRDM14 is critical for the reacquisition of potential pluripotency and successful epigenetic reprogramming during PGC specification [14,16]. Embryos with the complete deletion of *prdm14* generated impaired PGCs with reduced numbers and lower mobility [16]. In 11-week-old *prdm14* mutant females and males, germ cells in the ovaries and testes were completely lacking [16]. The simultaneous overexpression of *prdm1*, *prdm14*, and *tfap2c* could rapidly and efficiently induce epiblast-like cells (EpiLCs) to enter a PGC state in vitro [17]. Notably, *prdm14* alone was sufficient to direct the PGC state in EpiLCs [17]. In humans (*Homo sapiens*), PRDM1 functions as a repressor of somatic genes, which might allow SOX17, the key regulator of PGC-like cells’ (hPGCLCs) fate, to specify hPGCLCs [18]. The loss of *prdm14* significantly reduced the specification efficiency of hPGCLCs [19]. In chickens (*Gallus gallus*), both PRDM1 and PRDM14 play pivotal roles in the post-specification of PGC development and are involved in PGC self-renewal [20]. In crickets (*Gryllus bimaculatus*), *prdm1* is required for PGC formation and/or maintenance [21]. PRDM2 plays a potential tumor suppressor role in the formation of testicular germ cell tumors (TGCTs), which derive from PGCs [22]. PRDM8 participates in the regulation of mouse testis development [23]. And PRDM9 is a key determinant of meiotic recombination hotspots in both humans and mice [24,25]. Moreover, PRDMs are also involved in the proliferation and differentiation of embryonic stem cells [26,27,28,29], hematopoietic stem cells [30,31,32,33], and neurons [34,35,36,37,38,39]. Systematic identifications, expressive analyses, and functional studies of the PRDM gene family have been well conducted in vertebrates, but little information is available on invertebrates, especially on mollusks. This lack of information has prevented the elucidation of the evolution and conservation of the PRDM family, as well as the PGC development mechanisms in mollusks.

Mollusks are one of the most diverse groups in the animal kingdom; most of them are aquaculture species with important economic value. As they are an ancestral bilaterian group, studies on PGC specification in mollusks can help to elucidate the evolution of metazoan germ cell specification. Furthermore, understanding the molecular mechanisms of germ cells will facilitate the development of propagation control techniques and promote the aquaculture industry [40,41,42]. However, studies on the origin and development of PGCs in mollusks are scarce, and currently only three genes, *vasa*, *nanos*, and *piwi*, are used in related studies [7,8,9,43,44].

In the present study, we systematically identified and characterized PRDMs in *M. lateralis*, a promising model organism for bivalve mollusks. We then conducted a functional domain prediction, and sequence, phylogenetic, and expression level analyses of PRDMs. Moreover, we investigated the distribution of *Ml-prdm1* and *Ml-prdm14* transcripts in embryonic and larval stages. This study provided insights into the evolution of PRDMs and their potential involvement in PGC development in mollusks, laying a theoretical foundation for further functional investigations.

## 2. Materials and Methods

### 2.1. Identification of M. lateralis PRDMs

To identify PRDM members (Ml-PRDMs), we firstly searched the annotation files of the *M. lateralis* genome (unpublished data), and then combined the data with a protein-based sequence alignment and a domain-based sequence analysis. For the sequence alignment, orthologous PRDM sequences of vertebrates and invertebrates downloaded from databases including NCBI (https://www.ncbi.nlm.nih.gov/, accessed on 20 October 2022) and Uniprot (https://www.uniprot.org/, accessed on 23 October 2022) (Table S1) were used as query sequences for blastp using BioEdit 7.0.9 software [45] with an E-value threshold of 1 × 10^−5^. For the domain analysis, both hmmbuild and hmmsearch programs in HMMER v.3.0 [46] were used. Firstly, on the basis of sequence alignments with ClustalW (https://www.genome.jp/tools-bin/clustalw, accessed on 5 November 2022) among all Ml-PRDMs identified by blastp, the hmmbuild was carried out to generate a hidden Markov model (HMM) profile. The hmmsearch program (E-value < 1 × 10^−5^) was performed according to the HMM profiles for genome-wide domain analysis in *M. lateralis*. The candidate sequences obtained using these three methods were re-evaluated manually using CD-Search and Protein BLAST in the NCBI database with default parameters. Moreover, to better enrich the data on the PRDM gene family in shellfish for a more comprehensive phylogenetic analysis, the same identification procedure was applied to sequences from the *Chlamys farreri* genome [47].

### 2.2. PRDMs Sequence Analysis

The isoelectric point (pI) and molecular weight (Mw) were predicted using the Expasy Compute pI/MW tool (https://www.expasy.org/, accessed on 12 November 2022). The functional domains of Ml-PRDMs were visualized using IBS 1.0.3 [48] according to the prediction information from SMART (http://smart.embl-heidelberg.de/, accessed on 12 November 2022) and NCBI CD-Search. Multiple alignments of the PR domains among *M. lateralis*, *H. sapiens*, *M. musculus*, and *D. rerio* were performed using Clustal Omega on the EMBL-EBI website (https://www.ebi.ac.uk/, accessed on 12 November 2022), and the results were displayed using Jalview2.11.2.6 software [49].

### 2.3. Phylogenetic Analysis

The PR domains of sequences were extracted to perform a multiple sequence alignment using ClustalW with default parameters, and a phylogenetic tree was constructed using the maximum likelihood algorithm with default parameters in MEGA-X [50]. Finally, the tree was modified using the online software EvolView v3 (https://www.evolgenius.info/evolview, accessed on 10 March 2023).

### 2.4. Gene Expression Analysis of M. lateralis prdms

The HeatMap function of TBtools [51] was used to conduct heatmap and clustering analyses of *M. lateralis prdm* genes based on the published RNASeq datasets of four developmental stages: blastula, gastrula, trochophore, and D-shaped larvae (NCBI accession numbers SRR17520079–SRR17520090) [52].

### 2.5. Collection of Embryos and Larvae

Adult *M. lateralis* were provided by the MOE Key Laboratory of Marine Genetics and Breeding. Mature males and females were stimulated with seawater at 27 °C to lay eggs or to release sperms after cooling in a dry and dark environment for 1.5 h. Artificial insemination was then carried out based on a ratio of 1 egg to 10 sperms, and the fertilized eggs were incubated at 24 °C. Collected embryos and larvae were washed twice with PBS at room temperature and fixed with 4% paraformaldehyde overnight at 4 °C. Then, gradient dehydration was performed with serial methanol (25, 50, 75%, and 100%) after washing with PBS. Finally, the samples were stored in 100% methanol at −30 °C for whole-mount in situ hybridization analysis. Samples of the same developmental stages were also snap-frozen in liquid nitrogen and then stored at −80 °C for total RNA extraction.

### 2.6. Quantitative RT-PCR Analysis of Ml-prdm1 and Ml-prdm14

The expression patterns of *Ml-prdm1* and *Ml-prdm14* during early development stages were detected. Briefly, the total RNA of the fertilized egg, multicellular embryo, blastula, gastrula, trochophore, D-shaped larva, and umbo larva were extracted with the MicroElute Total RNA Kit (Omega, Norcross, GA, USA). The RNA’s integrity and quality were assessed by performing 1.2% agarose gel electrophoresis and spectrophotometry, and the first-strand cDNAs were reverse-transcribed using the SuperScript First-Strand Synthesis System (Invitrogen, Carlsbad, CA, USA). Quantitative RT-qPCR was conducted using the LightCycler 480 real-time fluorescence quantitative PCR instrument (Roche, Basel, Switzerland) with specific primers (Table 1), and *ef-1b* was utilized as an internal reference [52]. The 2^−ΔΔCt^ method [53] was used to calculate relative gene expression with three biological replicates and three technical replicates. The statistical analysis was tested using one-way ANOVA followed by Duncan’s test (SPSS 22.0, IBM Corp., Armonk, NY, USA), and the statistically significant difference was set as *p* < 0.05.

### 2.7. Whole-Mount In Situ Hybridization of Ml-prdm1 and Ml-prdm14

Specific fragments of *Ml-prdm1* and *Ml-prdm14* were amplified using gastrula cDNA and gene-specific primers (Table 2) containing T7 or SP6 promoter sequences. The resultant PCR products were used as templates for in vitro transcription to synthesize digoxigenin-labeled RNA probes according to the manufacturer’s instructions for the DIG RNA labeling kit (Roche, Mannheim, Germany). In addition, the labeled probes were precipitated using ethanol and lithium chloride, and dissolved in RNase-free water.

Whole-mount in situ hybridization was performed as previously described [54] with slight modifications. The rehydrated samples were treated with proteinase K (1 μg/mL for embryos and 4 μg/mL for trochophore larvae) at 37 °C for 20 min before incubation in TAE buffer. After treatment with acetic anhydride, samples were post-fixed in 4% paraformaldehyde for 30 min. Samples were pre-hybridized in hybridization buffer (50% deionized formamide, 5% dextran sulfate, 0.1% tween-20, 5 × SSC, 50 μg/mL heparin, 500 μg/mL yeast total RNA, 18 mM citric acid) at 60 °C for three hours, followed by being hybridized with 6 μg/mL denatured RNA probe at 60 °C overnight and then washed in gradient saline sodium citrate buffer at 60 °C. After treatment in blocking buffer for 1 h, samples were incubated with 1:2500 diluted alkaline phosphatase-conjugated digoxigenin antibody (Roche, Mannheim, Germany) overnight at 4 °C. Samples were finally incubated with NBT-BCIP in staining buffer (100 mM Tris-HCl, 100 mM NaCl, 10% PVA, 50 mM MgCl_2_, 0.1% Tween-20, pH 9.5) in darkness at 37 °C for color development.

## 3. Results

### 3.1. Identification and Sequence Analysis of M. lateralis Putative PRDMs

**Figure 1:**
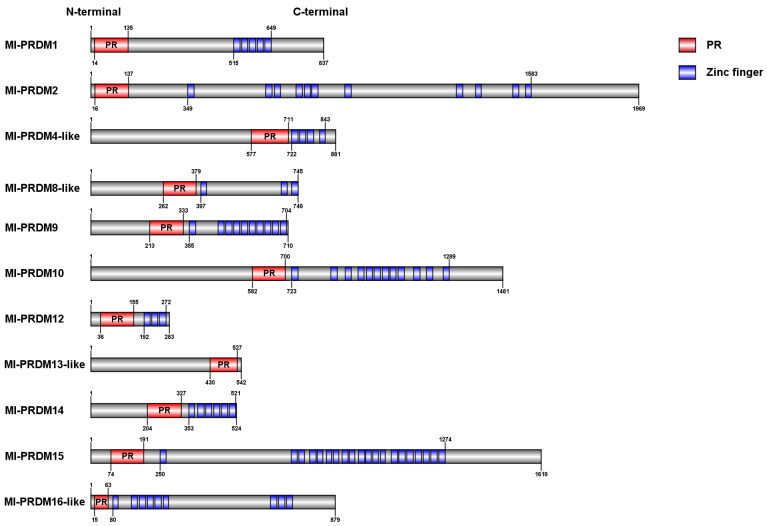
Conserved domain identification of PRDM proteins of *M. lateralis*.

Eleven members of the PRDM family were identified in the genome of *M. lateralis*. All of these Ml-PRDMs had one PR domain and several zinc fingers (0–20), and their protein lengths varied from 283 to 1969 aa (Figure 1; Table 3). The members identified with both SMART and NCBI CD-Search were named Ml-PRDM, whereas those recognized by only one algorithm were designated as Ml-PRDM-like. The predicted physical and chemical properties, molecular weights, and isoelectric points of all PRDM members were presented in Table 3.

**Figure 2:**
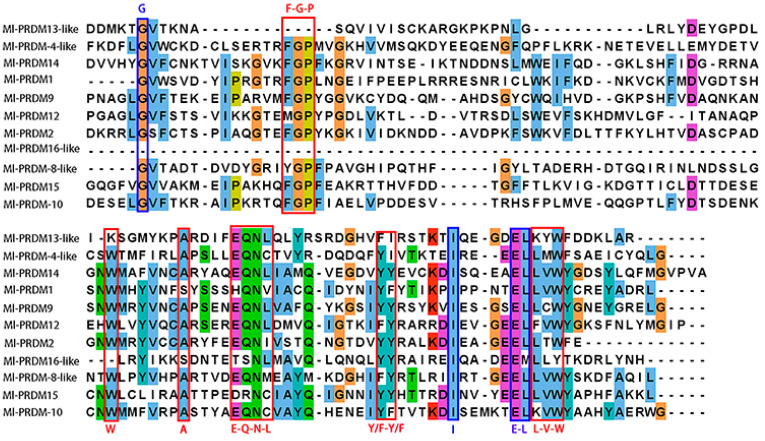
Multiple sequence alignment of the PR domains in *M. lateralis*. Blue boxes indicate shared conserved residues in the PR and SET domains, and red boxes indicate exclusively conserved residues in the PR domains.

**Figure 3:**
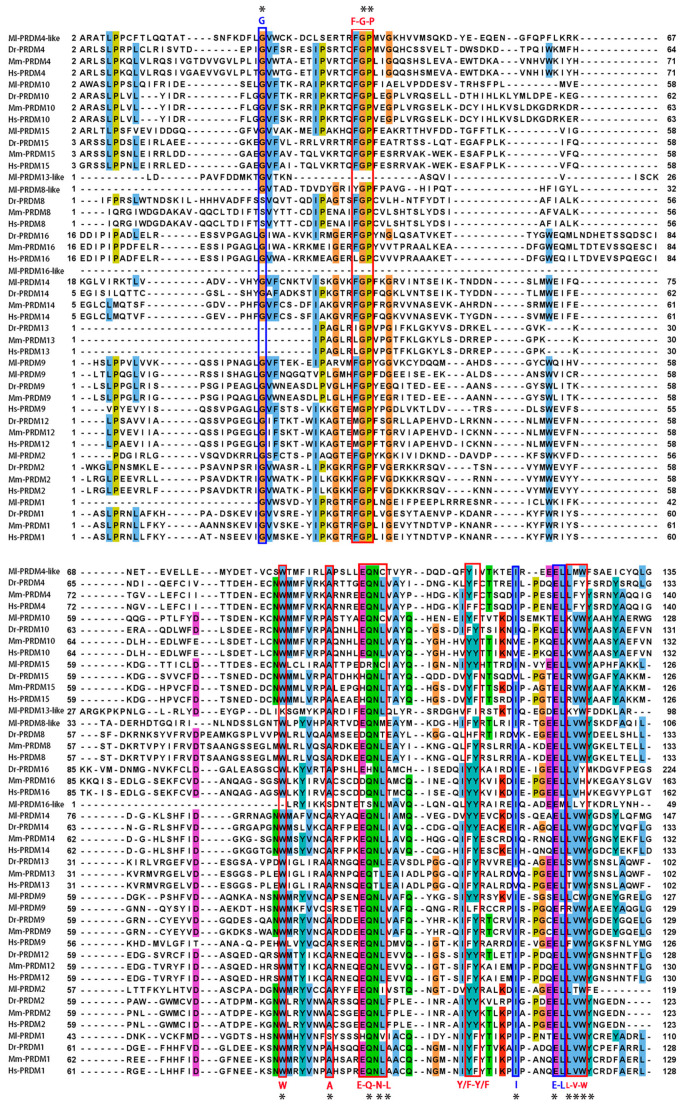
Multiple sequence alignment of the PR domains in different species. Asterisks indicate residues with more than 80% identity, blue boxes indicate shared conserved residues in the PR and SET domains, and red boxes indicate exclusively conserved residues in the PR domains.

Four highly conserved residues (G, I, and E-L) in both PR and SET domains [55] were observed in the PR domains of all the Ml-PRDMs, except for the Ml-PRDM16-like, of which the G and L were not conserved (Figure 2). In addition, six well-conserved and unique residues (W, A) and motifs (F-G-P, E-Q-N-L, Y/F-Y/F, L-V-W), which were almost exclusive to the PR domains [55,56,57], were partially or completely identified in Ml-PRDMs (Figure 2). Notably, most of these residues and motifs were less conserved in Ml-PRDM13-like and Ml-PRDM16-like proteins. A multiple sequence alignment of 44 sequences between *M. lateralis* and three vertebrates showed that the identity of 14 residues was higher than 80%, and there was a residue (E) exhibiting great conservation in all sequences (Figure 3).

### 3.2. Phylogenetic Analysis of Putative M. lateralis PRDMs

**Figure 4:**
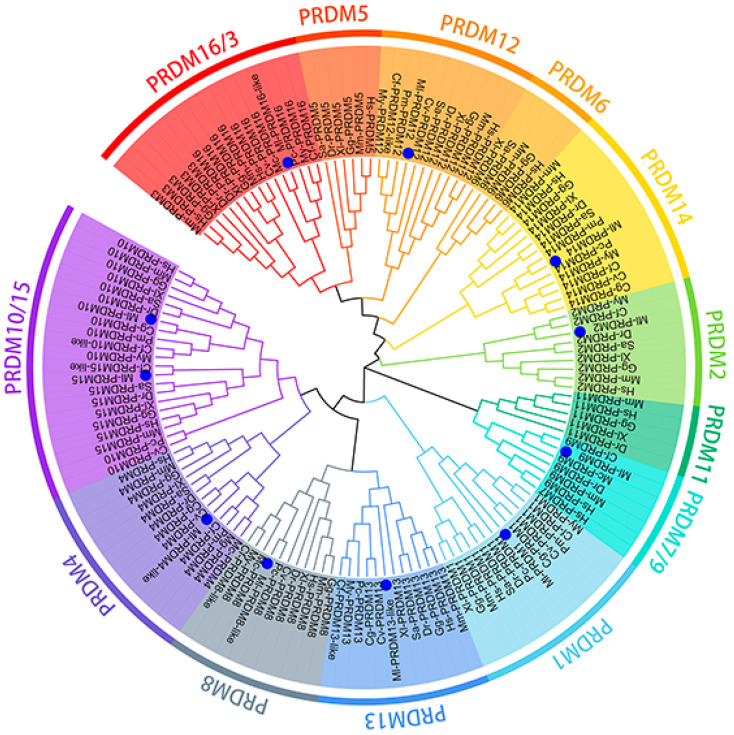
Phylogenetic analysis of M. lateralis PRDM proteins. Hs: *Homo sapiens*; Mm: *M. musculus*; Gg: *G. gallus*; Xl: *Xenopus laevis*; Dr: *Danio rerio*; Sa: *Sparus aurata*; My: *M. yessoensis*; Mc: *Mytilus coruscus*; Cv: *C. virginica*; Cg: *C. gigas*; Pm: *Pecten maximus*; Mg: *Mytilus galloprovincialis*; Pc: *Pomacea canaliculate*; Cf: *C. farreri*. Blue dots indicate M. lateralis PRDMs.

The putative PRDM proteins of *M. lateralis* were further confirmed using phylogenetic analysis (Figure 4). In the phylogenetic tree constructed, Ml-PRDM1 and Ml-PRDM14 displayed the closest phylogenetic relationships with other bivalves. Ml-PRDM9 and Ml-PRDM15 displayed the closest phylogenetic relationships with *C. farreri*. Ml-PRDM10 exhibited the closest clustering relationship with *Crassostrea gigas* and Ml-PRDM4 clustered with *Crassostrea virginica* and *C. gigas*. In short, *M. lateralis* PRDMs were closely clustered with different molluskan PRDMs and then grouped with vertebrate PRDMs in each family member clade.

### 3.3. Temporal Expression Pattern of Putative M. lateralis prdms during Early Development Stages

**Figure 5:**
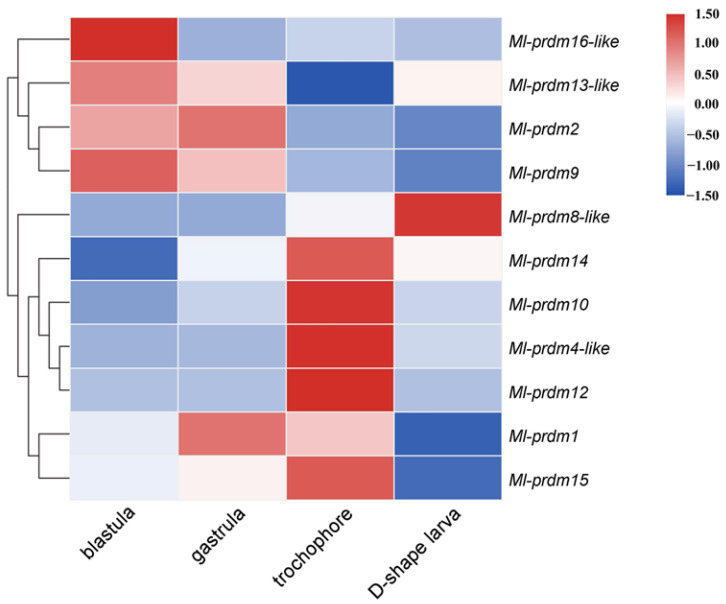
Expression profiles of *prdm* genes during early development stages of *M. lateralis*.

*M. lateralis prdms* exhibited dynamic expression patterns during embryonic and larval stages, which were obtained from the published RNASeq datasets (NCBI accession numbers SRR17520079–SRR17520090) (Figure 5). Three family members (*Ml-prdm9*, *Ml-prdm13-like*, *Ml-prdm16-like*) were highly expressed in the blastula; two family members (*Ml-prdm1*, *Ml-prdm2*) showed the highest expression levels in the gastrula stage; and the highest expression of five members (*Ml-prdm14*, *Ml-prdm10*, *Ml-prdm4-like*, *Ml-prdm12*, and *Ml-prdm15*) occurred in the trochophore larva stage, whereas only *Ml-prdm8-like* had the highest expression level in the D-shaped larva stage. According to the heatmap, the expression profiles of *Ml-prdm1* and *Ml-prdm14* exhibited a similar trend: the expression level initially increased and then subsequently decreased. However, the highest expression of *Ml-prdm1* occurred earlier than that of *Ml-prdm14*.

**Figure 6:**
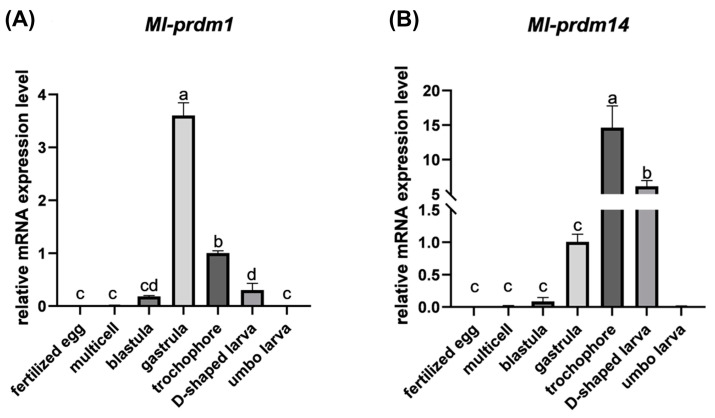
Expression of *Ml-prdm1* and *Ml-prdm14* during early development stages of *M. lateralis*. (**A**,**B**) represented *Ml-prdm1* and *Ml-prdm14*, respectively. Genes expression level of trochophore or gastrula was set as standard 1, respectively, and the expression levels in other stages were indicated as relative fold-change. All data were represented as mean ± SD (*n* = 3). Different letters indicated statistically significant differences (*p* < 0.05).

With the RT-qPCR analysis, it was shown that *Ml-prdm1* started to express at the multicellular stage and reached a peak value when developed to the gastrula stage. The expression decreased at the trochophore larva stage and declined sharply after that (Figure 6). A similar pattern could be seen in *Ml-prdm14*, with the exception that the trochophore larva occupied the highest expression level and the D-shaped larva was second to that.

### 3.4. Spatial Expression Pattern of M. lateralis prdm1 and prdm14 in Embryo and Larva

**Figure 7:**
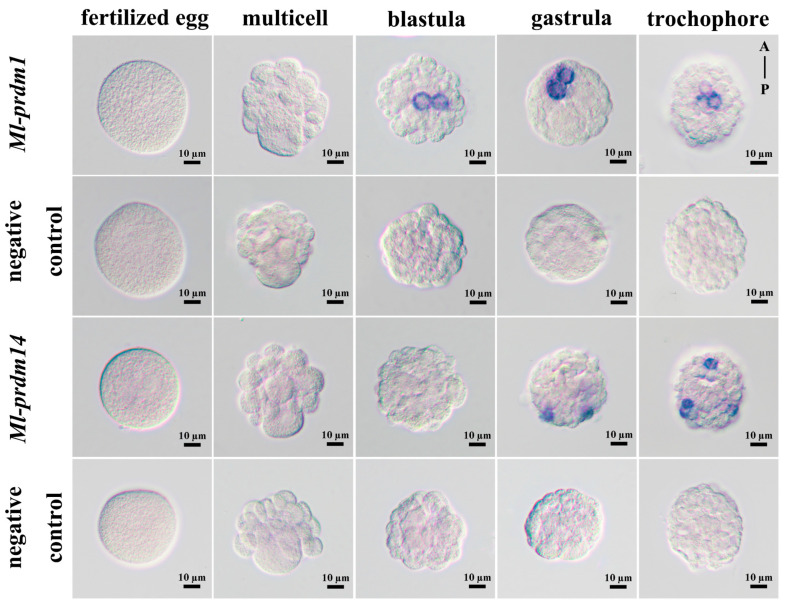
The localization of *Ml-prdm1* and *Ml-prdm14* transcripts in early development stages of *M. lateralis* detected by whole-mount in situ hybridization.

To reveal the potential relevance of PRDMs to PGC specification, the spatial distribution of *Ml-prdm1* and *Ml-prdm14* mRNA from fertilized egg to trochophore larva was investigated by carrying out whole-mount in situ hybridization. According to the results, no signals of *Ml-prdm1* or *Ml-prdm14* transcripts were detected in fertilized eggs and multicellular embryos. The signals of *Ml-prdm1* were initially detected in the blastula, but those of *Ml-prdm14* were observed firstly in the gastrula, which was consistent with the temporal expression pattern that *Ml-prdm14* exhibited, with higher expression in later stages compared to *Ml-prdm1*. The signals of *Ml-prdm1* were specifically concentrated in the cytoplasm of two adjacent cells in the blastula and gastrula (Figure 7 and Figure S1). And then, in the trochophore larva, *Ml-prdm1* transcripts were localized in three cells in the middle of the body, and the signals were still mainly located in the cytoplasm. In contrast, the positive signals of *Ml-prdm14* appeared as two or three dots scattered in cell clusters at the edge of the gastrula or trochophore larva, respectively (Figure 7). These two genes displayed quite distinct expression patterns, implying that they may have different functions.

## 4. Discussion

The number of *prdm* genes identified per species is significantly different, ranging from 2 in the sponge *Oscarella carmela* to 19 in teleosteans [55]. Such differences of gene numbers also exist within each main animal lineage [55]. In lophotrochozoans, 6 to 8 genes can be found in platyhelminthes, whereas in mollusks, the number of *prdm* genes ranges from 8 in *Pinctada fucata* to 13 in *C. gigas* [55]. In the present study, we identified 11 putative PRDMs in both the *M. lateralis* and *C. farreri* genomes. The identified Ml-PRDM13-like lacked zinc fingers in this study because of the non-integral nature of its DNA sequence in the genome. However, its deduced amino acid sequences and PR domain were conserved with the PRDM13 of other species, and this member was retained in the present study.

In vertebrates, several PRDM proteins have been shown to possess intrinsic HMTase activity similar to that of SET proteins [23,31,58,59,60,61], whereas others that lack this intrinsic activity can participate in epigenetic regulation by interacting with enzymes capable of methylating histones or catalyzing other post-translational modifications of chromatin [30,62,63,64]. Four highly conserved residues shared in the PR and SET domains as well as six well-conserved and unique residues and motifs that are exclusive to the PR domains [55,56,57] were identified in Ml-PRDMs (Figure 2). Furthermore, fourteen residues showed an identity of higher than 80% between *M. lateralis* and vertebrates (Figure 3). These conserved residues and motifs suggest that Ml-PRDMs may exert molecular functions similar to those of their vertebrate homologous proteins.

PRDMs are transcriptional factors that regulate many fundamental aspects of cellular differentiation [65]. Transcriptomic analysis revealed that *M. lateralis prdm* genes exhibited dynamic expression patterns during embryonic and larval development (Figure 5). It is worth mentioning that nearly half of the *M. lateralis prdm* genes had the highest expression levels in the trochophore larva, including *Ml-prdm4-like*, *Ml-prdm10*, *Ml-prdm12*, *Ml-prdm14*, and *Ml-prdm15*. In many mollusks, the trochophore stage is the beginning of neurogenesis [66,67,68,69]. It has been reported that *prdm4*, *prdm12*, and *prdm14* are involved in vertebrate neurological development [36,70,71,72]. Therefore, we can presume that these members of the PRDM gene family may have important functions in larval nervous system development.

It was speculated that *Ml-prdm1*-positive cells are putative PGCs or their progenitors in *M. lateralis*. PGCs in mollusks can be recognized in mesodermal tissues around the time of gastrulation [73,74]. In the transcriptomic and quantitative RT-PCR analysis results, we observed the highest expression of *Ml-prdm1* at the gastrula stage (Figure 5 and Figure 6), suggesting that *Ml-prdm1* may be involved in PGC formation in *M. lateralis*. The specific expression patterns of *prdm1* orthologs have been revealed in many organisms, including *Drosophila melanogaster* [75], *G. bimaculatus* [21], *Petromyzon marinus* [76], *Strongylocentrotus purpuratus* [77], *D. rerio* [78], *X. laevis* [79], *Ambystoma mexicanum* [80], *G. gallus* [81], and *M. musculus* [13,82], and *prdm1* was identified as the PGC marker in most of these animals. Usually, a subset of cells labeled by marker genes, such as *vasa* and *nanos*, are identified as putative PGCs or their precursors in mollusks. Although the expression patterns of these markers throughout embryonic development are not completely the same, the bivalve blastula and gastrula shared a similar subcellular localization of markers, which were restricted to two cells or cell clumps [7,8,9,43]. In the present study, *Ml-prdm1* exhibited a restricted expression in the embryos and larvae of *M. lateralis*. In the blastula and gastrula, we observed two hybridization signals restricted to only two cells (Figure 7). In the trochophore larva, hybridization signals specifically appeared in three cells without any signals elsewhere (Figure 7). The distribution of *Ml-prdm1* transcripts around the gastrula stage was remarkably similar to some of the putative PGC markers, especially *nanos*, in other bivalves [8,9]. In addition, *Ml-prdm1*-positive cells exhibited slow cell cycles from the blastula to the trochophore larva, and this was also consistent with observations in other mollusks in which PGCs were mitotically quiescent for a period [73,74]. Therefore, we hypothesized that *Ml-prdm1* is a candidate PGC marker gene.

In mice, the expression of *Ml-prdm1* preceded *Ml-prdm14* [16,83,84], and a similar expression pattern was detected in the present study. However, the expression of *prdm14* has been detected in presumptive *prdm1*-positive cells representing PGCs and their precursors in mice [16,85]. In contrast, the spatial expression patterns of these two genes in *M. lateralis* were completely different and did not seem to be co-expressed (Figure 7). It has been reported that *prdm14* in *G. bimaculatus* is absent, although *prdm1* has been shown to play an essential role in PGC development, indicating the different roles of *prdm14* [21]. Moreover, we failed to identify an ortholog of the vertebrate *tfap2c* gene either in the transcriptome or in the genome, which is another regulator cooperating with *prdm1* in the specification of murine PGCs [1,84]. Therefore, we assumed that *Ml-prdm14* was probably not expressed in *M. lateralis* PGCs and was dispensable for their specification. This distinction suggested the divergence of the molecular network for PGC formation between vertebrates and invertebrates. Further functional validations should be performed to elucidate the mechanisms of PGC formation in mollusks.

## 5. Conclusions

In the present study, we identified 11 PRDM gene family members in *M. lateralis* based on genome-wide screening. The functional domain compositions, sequence identities, phylogenetic relationships, and expression profiles of the *M. lateralis* PRDMs were uncovered. Furthermore, we investigated the distribution of *Ml-prdm1* and *Ml-prdm14* transcripts during the early developmental stages, ranging from fertilized eggs to trochophore larvae. The results suggested that *Ml-prdm1* may present functional conservation in terms of PGC specification similar to that in vertebrates. This study provides insights into the evolution of PRDMs in mollusks and offers clues for the further investigation of *prdm1* in the formation of PGCs in mollusks.

## Figures and Tables

**Table 1:** Primer sequences used for quantitative RT-qPCR.

Gene Name	Forward Primer (5′-3′)	Reverse Primer (5′-3′)
*Ml-prdm1*	AGGAGAGGAAATGTTACAAGCA	GGCAGGAGTAGGTGGAGTCTTA
*Ml-prdm14*	ACTGACGCCTCGGTTTTATC	GTCGGACTCGGTGTTTCTGA
*Ml-ef1b*	GGGCATTACTTCACTCTAAAT	TGTGCTATCTGAGGGTCTACT

**Table 2:** Primer sequences used for the synthesis of RNA probes of in situ hybridization.

Gene Name	Forward Primer (5′-3′)	Reverse Primer (5′-3′)
*Ml-prdm1*	ATTTAGGTGACACTATAGAAGCG ^1^ CATTTAAGAGCCCGAAGT	TAATACGACTCACTATAGGGAGACA ^2^ CATAGGAGGGTAAGGTGG
*Ml-prdm14*	ATTTAGGTGACACTATAGAAGCG ^1^ GATTGGTTTCCCATTTC	TAATACGACTCACTATAGGGAGACA ^2^ TCGTGCTCCTCTGACTT

^1^ The underlined sequence represents the SP6 promoter. ^2^ The underlined sequence represents the T7 promoter.

**Table 3:** Summary of *M. lateralis* PRDMs properties.

Protein	Protein Length	Domain Number		PR Domain Length	pI	Mw (Da)
		PR	Zinc Finger			
Ml-PRDM1	837	1	5	122	6.96	95,462.42
Ml-PRDM2	1969	1	11	122	6.04	221,527.46
Ml-PRDM4-like	881	1	4	135	5.74	98,698.34
Ml-PRDM8-like	746	1	3	118	7.96	84,010.47
Ml-PRDM9	710	1	10	121	8.54	81,667.44
Ml-PRDM10	1481	1	12	119	6.25	165,430.32
Ml-PRDM12	283	1	3	120	9.2	32,469.99
Ml-PRDM13-like	542	1	0	98	8.54	60,496.27
Ml-PRDM14	524	1	6	124	8.91	59,197.42
Ml-PRDM15	1619	1	20	118	5.96	181,519.82
Ml-PRDM16-like	879	1	9	49	6.76	98,759.51

## Data Availability

The data presented in this study are available in this article or in the Supplementary Materials.

## Supplementary Materials

The following supporting information can be downloaded at: https://www.mdpi.com/article/10.3390/biology12081059/s1, Table S1: Accession numbers of PRDM protein sequences. Figure S1: The visualization of *Ml-prdm1* expressing cells in early development stages of *M. lateralis*.
